# Comparable performance of machine learning algorithms in predicting readmission and complications following total joint arthroplasty with external validation

**DOI:** 10.1186/s42836-023-00208-0

**Published:** 2023-11-08

**Authors:** Hashim J. F. Shaikh, Mina Botros, Gabriel Ramirez, Caroline P. Thirukumaran, Benjamin Ricciardi, Thomas G. Myers

**Affiliations:** Department of Orthopaedics and Physical Performance, University of Rochester Medical Center, 601 Elmwood Ave, Rochester, NY 14642 USA

**Keywords:** Machine learning, External validation, Total joint arthroplasty, Database, Complications

## Abstract

**Background:**

The purpose of the study was to use Machine Learning (ML) to construct a risk calculator for patients who undergo Total Joint Arthroplasty (TJA) on the basis of New York State Statewide Planning and Research Cooperative System (SPARCS) data and externally validate the calculator on a single TJA center.

**Methods:**

Seven ML algorithms, i.e., logistic regression, adaptive boosting, gradient boosting (Xg Boost), random forest (RF) classifier, support vector machine, and single and a five-layered neural network were trained on the derivation cohort. Models were trained on 68% of data, validated on 15%, tested on 15%, and externally validated on 2% of the data from a single arthroplasty center.

**Results:**

Validation of the models showed that the RF classifier performed best in terms of 30-d mortality AUROC (Area Under the Receiver Operating Characteristic) 0.78, 30-d readmission (AUROC 0.61) and 90-d composite complications (AUROC 0.73) amongst the test set. Additionally, Xg Boost was found to be the best predicting model for 90-d readmission and 90-d composite complications (AUC 0.73). External validation demonstrated that models achieved similar AUROCs to the test set although variation occurred in top model performance for 90-d composite complications and readmissions between our test and external validation set.

**Conclusion:**

This was the first study to investigate the use of ML to create a predictive risk calculator from state-wide data and then externally validate it with data from a single arthroplasty center. Discrimination between best performing ML models and between the test set and the external validation set are comparable.

**Level of Evidence:**

III.

## Introduction

Orthopaedic procedures involving total hip and knee replacement categorically account for the largest annual expenditures by Medicare by specialty and procedures, respectively [1–3]. Consequently, the Centers for Medicare and Medicaid Services (CMS) instituted the Comprehensive Care for Joint Replacement (CJR) model. CJR reduced costs and readmissions while potentially exacerbating disparities in the access to total joint arthroplasty (TJA) in some but not all centers [4–6]. The potential disparities in TJA access may have arisen, in part, due to an aversion to certain high-risk patient populations by some centers participating in the CJR model [7]. High-risk TJA patients are associated with increased resource demands, increased costs, and decreased reimbursement [8–10]. Therefore, the ability to identify patients who may be at a higher risk of poor outcomes following TJA may allow for resource reallocation to reduce this risk.

Artificial intelligence (AI) and machine learning (ML) algorithms can pattern the interactions of variables within datasets to create predictive models [11, 12]. Machine learning in medicine and orthopaedics has begun to take hold in the past five years, as manifested by publications specifically related to ML in TJA [13]. Successful creation of accurate preoperative risk calculators using ML algorithms would let providers preoperatively identify patients who may be at increased risk for poor outcomes following TJA. The ability to preoperatively predict increased risk would provide two potential opportunities. The first is the optimization of modifiable risk factors that may lower the cost of care [14]. The second is the ability to risk stratify reimbursement and reconcile current disparities in TJA access.

The performance of ML prediction models is directly proportional to the degree of data quality and quantity. Previous ML studies in TJA prediction models have been conducted using both “big” national databases and single center registries to predict outcomes, including discharge disposition, complications, mortality, satisfaction, and minimally important clinical differences following TJA [15–18]. While utilization of “big” databases provides ML algorithms access to the large patient volumes and variables necessary for ML algorithm training and model accuracy, these datasets present challenges. A specific challenge with “big” datasets is that variables, such as population diversity, changes in medical practice patterns, healthcare policy differences between states, and geographic data used to train the ML algorithms, create models that to date are of questionable clinical utility when applied to any specific patient. This point was highlighted by Harris et al., who concluded that “models previously developed with VASQIP (Veterans Affairs Surgical Quality Improvement Program) data had poor accuracy when externally validated with NSQIP (National Surgical Quality Improvement Program) data, suggesting that they should not be used outside the context of the Veterans Health Administration” [17]. Single center registries are a potential alternative to train ML algorithms using datasets that more closely represent the TJA patients who will prospectively undergo risk factor stratification with the trained model. While single-center registries overcome the limitations associated with “big” datasets, they may not contain the necessary patient volumes required to train ML algorithms, ultimately leading to models suffering from the same questionable clinical utility as those derived from “big” data. As a case in point, many authors do not achieve an AUROC > 0.8. and we are aware of only one attempt to externally validate a model [17].

Therefore, to overcome some of the limitations previously mentioned in both single center registries and many “big” datasets, we aimed to use the New York State (NYS) Statewide Planning and Research Cooperative System (SPARCS) all-payer administrative database to train ML models for 30- and 90-day readmissions and 90-day composite complications following TJA. For this particular study, the SPARCS dataset stands out as a superior resource among large healthcare datasets due to its comprehensive and consistent data collection methodology. Unlike other datasets that rely on random sampling or limited enrollment, SPARCS mandates that every healthcare facility (inpatient and outpatient) within NYS consistently contribute its data. This approach ensures the inclusion of the entirety of NYS’s healthcare landscape, a crucial factor for research and analysis conducted within the geographic region of our center [17]. One of the key strengths of SPARCS lies in its ability to capture the entire population. By encompassing data from all healthcare facilities in the state, it minimizes the risk of sampling bias that can be introduced by random sampling or enrollment-based datasets (e.g., a patient receiving surgery in hospital A is included in the database but is readmitted to hospital B not included in the database). The potential for substantial demographic differences across states is a concern often encountered when working with datasets that exclude certain hospitals or regions. NYS SPARCS effectively mitigates this concern, as it offers a comprehensive snapshot of the healthcare experiences and outcomes within the entire New York State population. Because SPARCS has coded individual hospitals within its all-payer dataset, we would identify and externally validate 90-day complications, 30-day mortality, and 30- & 90-day readmissions of our primary single arthroplasty center, against a model trained from SPARCS data. We hypothesized that externally validated models from our primary arthroplasty center (PAC) would demonstrate similar performance compared to internally validated models.

## Methods

### Data source

This study was a retrospective review of the NYS SPARCS database. Developed in 1979, the SPARCS database is a de-identified, all-payer, patient-specific database maintained by the NYS Department of Health. State legislature requires that all NYS hospitals, ambulatory surgery centers, emergency departments, outpatient hospital-extension clinics, as well as diagnostic and treatment centers should periodically report data to compile the extensive database. Information reported includes patient-level data on characteristics (e.g., demographics, BMI, etc.), diagnostic and surgical codes, services provided, charges incurred and hospital as well as provider identifiers. By assigning each patient a unique identifier, the database can provide reliable data with a high degree of continuity of an individual patient’s care across hospital systems statewide (e.g., readmissions). More information can be found at https://www.health.ny.gov/statistics/sparcs/.

### Study population

Following approval by our Institutional Review Board, the SPARCS database was queried for all patients who underwent elective total hip or knee arthroplasty between 1 January 2012 and 31 December 2016. We used the Centers for Medicare and Medicaid Services (CMS) algorithm and ICD-9 and ICD-10 procedure codes for identifying the cohort of interest [18]. We employed ICD-9 and ICD-10 diagnosis and procedure codes specified by CMS to exclude patients undergoing joint replacements for fractures, revision/resurfacing/removal of implanted devices or prostheses, mechanical complications, malignant neoplasms, and partial hip replacements. The benefit of the SPARCS database is the comprehensive catchment of all cases performed in New York State regardless of payer. Additionally, each patient has a unique identifier allowing the patient to be tracked across hospital readmissions at different institutions within the state. Patient-level data were linked to the American Hospital Association (AHA) Annual Survey database to obtain hospital characteristics (community or teaching hospital, hospital size, urban/rural, geographic location, and hospital ownership) for inclusion in the models.

### Explanatory variables/predictors

Baseline demographics were collected, including age, sex, race, ethnicity, zip code, anatomic site (hip or knee), hospital identifier, admission source, diagnosis code, discharge destination, payer source (Medicare versus commercial insurance), year of surgery, and method of anesthesia. Comorbidity indicators were defined using the Elixhauser’s Comorbidity Index (Table 1).

**Table 1:** Descriptive characteristics

**Descriptive**		
***N* (%)**	247,875	(100.00)
**Sex, *N* (%)**		
Female	151,086	(60.95)
Male	96,789	(39.05)
**Race, *N* (%)**		
White	192,404	(77.62)
Black	23,522	(9.49)
Other	31,949	(12.89)
**Primary Payor,* N* (%)**		
Private	117,455	(47.38)
Medicare	111,824	(45.11)
Medicaid	5,994	(2.42)
Other Federal	1,100	(0.44)
Other	11,502	(4.64)
**Admission Type, *N* (%)**		
Emergent	2,751	(1.11)
Urgent	2,793	(1.13)
Elective	242,211	(97.71)
Other	120	(0.05)
**Source of Admission, *N* (%)**		
Health Facility	38,944	(15.71)
Non-Health Facility	208,931	(84.29)
**Anesthesia Method, *N* (%)**		
No Anesthesia	75,426	(30.43)
General Anesthesia	73,713	(29.74)
Regional Anesthesia	88,616	(35.75)
Other Anesthesia	10,120	(4.08)
**Discharge Destination, *N* (%)**		
Home	40,037	(16.15)
Home w/ Home Health Agency	101,769	(41.06)
Inpatient Rehab Facility	25,891	(10.45)
Skilled Nursing Facility	78,915	(31.84)
All others	1,263	(0.51)
**Elixhauser Comorbidity Sum, *N* (%)**		
0	33,574	(13.54)
1	64,550	(26.04)
2	67,244	(27.13)
3	44,909	(18.12)
4	22,615	(9.12)
5	9,468	(3.82)
6	3,619	(1.46)
7	1,272	(0.51)
8	424	(0.17)
9	145	(0.06)
10	48	(0.02)
11	X	(0.00)
12	X	(0.00)
**Congestive Heart Failure, *N* (%)**	5,937	(2.40)
**Cardiac Arrhythmias, *N* (%)**	27,273	(11.00)
**Valvular Disease, *N* (%)**	11,323	(4.57)
**Pulmonary Circulation Disorders, *N* (%)**	Present	2460
**Peripheral Vascular Disorders, *N* (%)**	5,084	(2.05)
**Hypertension, Uncomplicated, *N* (%)**	146,559	(59.13)
**Paralysis, *N* (%)**	233	(0.09)
**Other Neurological Disorders, *N* (%)**	4,731	(1.91)
**Chronic Pulmonary Disease, *N* (%)**	39,561	(15.96)
**Diabetes, Uncomplicated, *N* (%)**	40,590	(16.38)
**Diabetes, Complicated, *N* (%)**	3,937	(1.59)
**Hypothyroidism, *N* (%)**	37,569	(15.16)
**Renal Failure, *N* (%)**	10,153	(4.10)
**Liver Disease, *N* (%)**	3,136	(1.27)
**Peptic Ulcer Disease Excluding Bleeding, *N* (%)**	1,014	(0.41)
**Lymphoma, *N* (%)**	649	(0.26)
**Metastatic Cancer, *N* (%)**	204	(0.08)
**Solid Tumor Without Metastasis, *N* (%)**	1,174	(0.47)
**Rheumatoid Arthritis/Collagen Vascular, *N* (%)**	10,955	(4.42)
**Coagulopathy, *N* (%)**	5,396	(2.18)
**Obesity, *N* (%)**	75,430	(30.43)
**Weight Loss, *N* (%)**	421	(0.17)
**Fluid and Electrolyte Disorders, *N* (%)**	21,941	(8.85)
**Blood Loss Anemia,* N* (%)**	952	(0.38)
**Deficiency Anemia, *N* (%)**	3,136	(1.27)
**Alcohol Abuse, *N* (%)**	2,960	(1.19)
**Drug Abuse, *N* (%)**	2,652	(1.07)
**Psychoses,* N* (%)**	991	(0.40)
**Depression,* N* (%)**	31,071	(12.53)
**Hypertension, Complicated, *N* (%)**	9,946	(4.01)

X represents a size that consists of less than ten individuals

### Outcomes

Primary outcomes of interest included 90-day complications, 30-day mortality, and 30- & 90-day all-cause readmissions following total hip and knee arthroplasty. Complications were defined by the following criteria: (1) acute myocardial infarction, pneumonia, or sepsis/septic shock occurring during the index admission or within a subsequent admission occurring within 7 days of the beginning of the index admission, (2) surgical site bleeding or pulmonary embolism during the index or subsequent admission taking place within 30 days of the start of the first admission (3) death during the index admission or within 30 days from index admission, (4) or mechanical complication, periprosthetic joint or surgical wound complication occurring within the index or subsequent admission occurring within 90 days from the start of the index admission.

### Statistical analysis

**Fig. 1:**
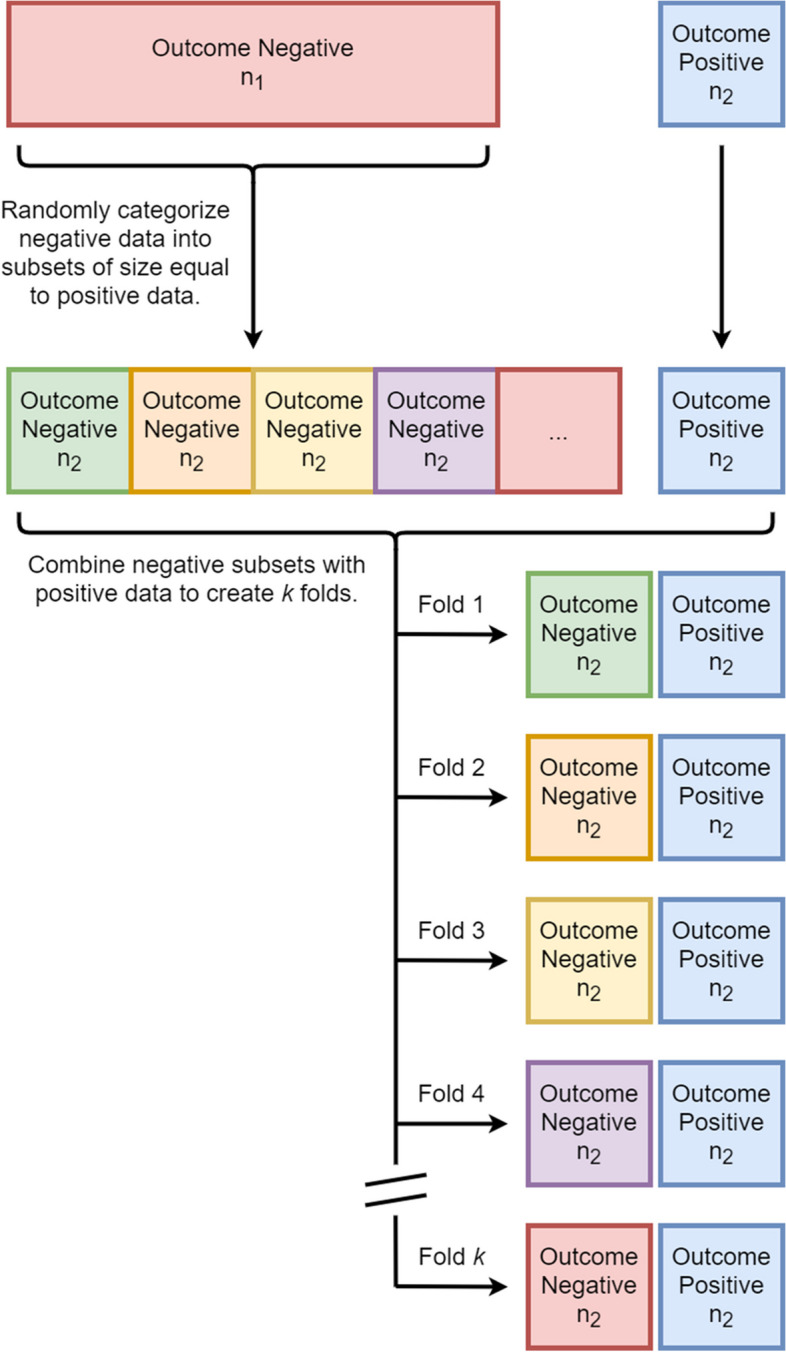
The negative outcome observations (e.g., did not have readmission) were randomly assigned to subsets equal to the number of positive outcome observations to address low incidence rates and technical limitations. Each negative outcome subset was combined with the positive outcome observations and used to one model instance

The dataset encompassing data from 3 January 2012 and 30 September 2016 was subdivided randomly, without replacement, into training (68%) validation (15%) and testing (15%) data sets. Finally, for external validation, *n* = 6000 (2%) TJA patients were identified within the SPARCS database from our PAC, using the hospital identifier, between 3 January 2012 and 30 September 2016. Normalization of continuous variables and one-hot encoding of categorical variables was performed after exclusively assigning each observation to a data set. The seven ML algorithms included: logistic regression (LR), adaptive boosting (AB), gradient boosting (Xg Boost), random forest (RF) classifier, support vector machine (SVM), a 1-layer neural network (NN), and a 5-layered NN. For the training data, the negative outcome observations (e.g., did not have a readmission) were randomly assigned to subsets equal to the number of positive outcome observations. For each preparation instance, parameters were optimized using a 5-fold cross-validated grid-search method to reduce over-fitting and enhance the generalizability of each model instance (Fig. 1). Each classifier was then validated on raw data, and classifier weights were readjusted upon calibration. Model weights were then fixed for each classifier variable and tested on the remaining non-trained SPARCS data. Finally, we externally validated the models with patient information from our PAC.

### Model evaluation

Discrimination refers to a model’s ability to distinguish between cases and non-cases and this is typically expressed in terms of accuracy, recall, precision, and AUROC. Accuracy is the number of correct model predictions and overall predictions [19]. The recall (sensitivity) of a model refers to its ability to correctly predict positive values out of the total number of all positive values (true positives and false negatives) in the dataset. The precision of a model measures the positive predictive value, essentially determining which outcomes are truly positive when compared against all predicted positives (true positives and false positives) [20]. The AUROC demonstrates the relationship between recall and the false positive rate (FPR). The FPR is defined as the number of incorrectly predicted positive outcomes overall outcomes that are actually negative (true negatives and false positives) [21, 22]. For a binary classification, such as a complication, each point’s location on the AUROC is found by assessing a variety of thresholds for sorting of *yi* in the positive or negative class. The top left corner of the curve is an ideal case with 100% of positive values correctly classified and 0% of positive values incorrectly predicted at 0. The goal for models, therefore, is to maximize the true positive rate while minimizing the FPR, the larger the area under the AUROC the better the model. Predictive modeling development and testing were performed under guidelines set forth by Transparent Reporting of a multivariable prediction model for Individual Prognosis or Diagnosis (TRIPOD) guidelines and the Guidelines for Developing and Reporting Machine Learning Models in Biomedical Research were followed for this analysis [19, 20].

## Results

### Baseline characteristics

A total of 247,875 patients were included in the cohort. The average age of the group was 65.4 ± 10.7 years and 60% of patients were female (Table 1). The complications included pulmonary embolism (0.56%), mechanical complication (0.49%) and pneumonia (0.39%) (Table 2). In reference to the entirety of the data set, 168,555 (68.0%) were segmented for training, 37,182 (15.0%) for validation and calibration, 37,182 (15.0%) for testing, and 6000 (2.00%) patients were included in the external validation set from our PAC.

**Table 2:** Complication characteristics

**Complication**		
**Acute Myocardial Infarction, *N* (%)**		
No	247,577	(99.88)
Yes	298	(0.12)
**Pneumonia, *N* (%)**		
No	246,910	(99.61)
Yes	965	(0.39)
**Sepsis/septicemia/shock, *N* (%)**		
No	247,405	(99.81)
Yes	470	(0.19)
**Surgical Site Bleeding, *N* (%)**		
No	247,765	(99.96)
Yes	110	(0.04)
**Pulmonary Embolism, *N* (%)**		
No	246,475	(99.44)
Yes	1,400	(0.56)
**Mechanical Complication, *N* (%)**		
No	246,667	(99.51)
Yes	1,208	(0.49)
**Periprosthetic Joint Infection/Wound Infection, *N* (%)**		
No	247,453	(99.83)
Yes	422	(0.17)

### Data balancing

The incidence rates of our test set for 90-day composite complications, 30-day mortality, and 30- & 90-day readmission, were 1.88%, 0.10%, 3.07%, and 5.15%, respectively. These rates were nearly identical for the training, validation and test set out to the hundredth decimal point. As for our PAC, the percentages for 90-day composite complications, and 30-day mortality, 30- & 90-day readmission were 1.33%, 0.13%, 2.94%, and 0.41% respectively. The negative outcome observations (e.g., did not have readmission) were randomly assigned to subsets equal to the number of positive outcome observations to address low incidence rates and technical limitations. Each negative outcome subset was combined with the positive outcome observations and used to one model instance.

### Testing data

After training and validating the models (Tables 3 and 4), testing on the untrained data (Table 5) showed RF classifier established the highest level of discrimination for 90-day complications (AUROC 0.73, 95CI 0.73–0.74), 30-day mortality (AUROC 0.78; 95CI 0.77–0.78), and 30-day readmission (AUROC 0.61; 95CI 0.60–0.61). Xg Boost demonstrated the best performance for 90-day hospital readmission (AUROC 0.73; 95CI 0.72–0.73).

**Table 3:** Model training on 68% of the dataset

		**Accuracy**				**Recall**				**Precision**				**AUROC**			
**Outcome**	**Model**	**M**	**SD**	**LB**	**UB**	**M**	**SD**	**LB**	**UB**	**M**	**SD**	**LB**	**UB**	**M**	**SD**	**LB**	**UB**
**90-D Composite Complications**	**AB**	0.86	0.001	0.86	0.86	0.61	0.001	0.61	0.61	0.08	0.000	0.08	0.08	0.74	0.000	0.74	0.74
	**Xg Boost**	0.74	0.002	0.74	0.75	0.77	0.003	0.77	0.78	0.003	0.000	0.003	0.003	0.76	0.001	0.76	0.76
	**LR**	0.81	0.01	0.79	0.83	0.38	0.004	0.37	0.39	0.07	0.002	0.06	0.07	0.60	0.002	0.60	0.61
	**RF**	0.80	0.002	0.80	0.81	0.41	0.001	0.41	0.42	0.11	0.000	0.11	0.11	0.62	0.000	0.62	0.62
	**SVM**	0.63	0.01	0.63	0.63	0.61	0.01	0.61	0.61	0.03	0.001	0.03	0.03	0.62	0.00	0.62	0.62
	**1 Layer NN**	0.88	0.21	0.87	0.89	0.23	0.28	0.21	0.25	0.05	0.10	0.04	0.05	0.56	0.07	0.56	0.57
	**5 Layer NN**	0.88	0.21	0.87	0.90	0.23	0.28	0.21	0.24	0.04	0.10	0.04	0.05	0.56	0.07	0.56	0.57
**30-D Mortality**	**AB**	0.80	0.001	0.80	0.80	0.99	0.002	0.98	0.99	0.07	0.001	0.07	0.07	0.87	0.001	0.87	0.88
	**Xg Boost**	0.74	0.001	0.74	0.75	0.93	0.003	0.92	0.93	0.004	0.000	0.004	0.004	0.84	0.002	0.83	0.84
	**LR**	0.62	0.01	0.61	0.63	0.941	0.02	0.89	0.99	0.07	0.002	0.06	0.07	0.77	0.01	0.75	0.80
	**RF**	0.65	0.001	0.65	0.65	1.000	0.00	1.00	1.00	0.12	0.000	0.12	0.13	0.81	0.001	0.81	0.81
	**SVM**	0.66	0.10	0.66	0.67	0.72	0.08	0.72	0.73	0.00	0.001	0.00	0.00	0.69	0.06	0.69	0.70
	**1 Layer NN**	0.89	0.22	0.88	0.91	0.34	0.37	0.31	0.36	0.00	0.01	0.00	0.00	0.61	0.13	0.60	0.62
	**5 Layer NN**	0.89	0.22	0.88	0.91	0.33	0.36	0.30	0.35	0.00	0.01	0.00	0.00	0.61	0.13	0.60	0.62
**30-D Readmission**	**AB**	0.88	0.001	0.88	0.88	0.59	0.001	0.59	0.59	0.09	0.000	0.09	0.09	0.74	0.000	0.74	0.74
	**GB**	0.95	0.000	0.95	0.95	0.53	0.000	0.53	0.54	0.01	0.000	0.01	0.01	0.74	0.000	0.74	0.74
	**LR**	0.85	0.001	0.85	0.85	0.34	0.001	0.34	0.34	0.07	0.000	0.07	0.07	0.60	0.000	0.60	0.60
	**RF**	0.82	0.001	0.81	0.82	0.39	0.001	0.38	0.39	0.11	0.000	0.11	0.12	0.61	0.000	0.61	0.61
	**SVM**	0.61	0.02	0.60	0.63	0.60	0.01	0.59	0.60	0.05	0.003	0.05	0.05	0.61	0.01	0.60	0.61
	**1 Layer NN**	0.87	0.20	0.86	0.88	0.16	0.24	0.15	0.18	0.03	0.03	0.03	0.03	0.53	0.03	0.53	0.53
	**5 Layer NN**	0.87	0.20	0.86	0.89	0.16	0.24	0.15	0.18	0.03	0.04	0.03	0.03	0.53	0.03	0.53	0.53
**90-D Readmission**	**AB**	0.88	0.001	0.88	0.88	0.99	0.000	0.99	0.99	0.13	0.001	0.13	0.14	0.93	0.000	0.93	0.94
	**GB**	0.77	0.001	0.77	0.78	0.95	0.002	0.94	0.95	0.004	0.000	0.004	0.004	0.86	0.001	0.86	0.86
	**LR**	0.80	0.01	0.78	0.83	0.99	0.000	0.99	0.99	0.14	0.01	0.13	0.15	0.89	0.01	0.88	0.91
	**RF**	0.79	0.01	0.76	0.81	0.99	0.000	0.99	0.99	0.20	0.01	0.18	0.21	0.88	0.01	0.87	0.90
	**SVM**	0.61	0.04	0.59	0.62	0.62	0.03	0.61	0.63	0.08	0.01	0.08	0.08	0.61	0.02	0.60	0.62
	**1 Layer NN**	0.86	0.20	0.84	0.87	0.16	0.24	0.15	0.18	0.05	0.06	0.05	0.06	0.53	0.03	0.53	0.53
	**5 Layer NN**	0.86	0.20	0.85	0.87	0.16	0.24	0.14	0.17	0.05	0.06	0.05	0.06	0.53	0.03	0.52	0.53

*LR* Logistic regression, *AB* Adaptive boosting, *Xg Boost* gradient boosting, *RF* Random forest, *SVM* Support vector machine, *NN* a 1-layer neural network and a 5 layered NN

**Table 4:** Validation and calibration on 15% of untrained data

		**Accuracy**				**Recall**				**Precision**				**AUROC**			
**Outcome**	**Model**	**M**	**SD**	**LB**	**UB**	**M**	**SD**	**LB**	**UB**	**M**	**SD**	**LB**	**UB**	**M**	**SD**	**LB**	**UB**
**90-D Composite Complications**	**AB**	0.86	0.001	0.86	0.87	0.57	0.001	0.57	0.58	0.08	0.000	0.07	0.08	0.72	0.14	0.000	0.13
	**Xg Boost**	0.74	0.002	0.74	0.75	0.65	0.002	0.64	0.65	0.003	0.000	0.003	0.003	0.70	0.01	0.000	0.01
	**LR**	0.81	0.008	0.79	0.83	0.35	0.008	0.34	0.37	0.06	0.001	0.061	0.067	0.59	0.107	0.002	0.10
	**RF**	0.80	0.001	0.80	0.81	0.40	0.001	0.39	0.40	0.11	0.000	0.113	0.114	0.62	0.18	0.001	0.17
	**SVM**	0.63	0.009	0.63	0.64	0.59	0.006	0.58	0.59	0.03	0.001	0.03	0.03	0.61	0.01	0.61	0.61
	**1 LAYER NN**	0.88	0.21	0.87	0.89	0.23	0.28	0.21	0.24	0.04	0.060	0.03	0.04	0.56	0.07	0.56	0.56
	**5 LAYER NN**	0.88	0.21	0.87	0.90	0.22	0.28	0.20	0.24	0.04	0.07	0.03	0.04	0.56	0.07	0.55	0.56
**30-D Mortality**	**AB**	0.76	0.002	0.75	0.77	0.66	0.002	0.65	0.66	0.05	0.000	0.050	0.051	0.71	0.09	0.001	0.09
	**Xg Boost**	0.74	0.001	0.74	0.75	0.67	0.002	0.66	0.67	0.003	0.000	0.003	0.003	0.70	0.005	0.000	0.09
	**LR**	0.60	0.004	0.59	0.61	0.61	0.003	0.60	0.62	0.05	0.000	0.046	0.047	0.61	0.09	0.001	0.085
	**RF**	0.61	0.001	0.61	0.62	0.62	0.001	0.61	0.62	0.08	0.000	0.079	0.080	0.62	0.14	0.000	0.14
	**SVM**	0.09	0.66	0.67	0.68	0.72	0.07	0.72	0.73	0.00	0.000	0.00	0.00	0.69	0.03	0.69	0.70
	**1 LAYER NN**	0.89	0.22	0.88	0.91	0.30	0.34	0.28	0.32	0.00	0.006	0.00	0.00	0.60	0.12	0.59	0.60
	**5 LAYER NN**	0.89	0.22	0.88	0.91	0.29	0.34	0.27	0.31	0.00	0.006	0.00	0.00	0.59	0.12	0.59	0.60
**30-D Readmission**	**AB**	0.88	0.001	0.88	0.88	0.57	0.001	0.56	0.57	0.09	0.000	0.087	0.089	0.73	0.153	0.000	0.15
	**Xg Boost**	0.95	0.000	0.95	0.95	0.33	0.001	0.32	0.33	0.01	0.000	0.007	0.007	0.64	0.01	0.000	0.01
	**LR**	0.84	0.001	0.84	0.85	0.32	0.002	0.31	0.32	0.07	0.000	0.069	0.070	0.59	0.11	0.000	0.11
	**RF**	0.82	0.001	0.81	0.82	0.39	0.002	0.38	0.39	0.12	0.000	0.11	0.12	0.62	0.18	0.001	0.18
	**SVM**	0.62	0.03	0.61	0.63	0.60	0.009	0.60	0.61	0.05	0.003	0.05	0.05	0.61	0.02	0.60	0.62
	**1 LAYER NN**	0.87	0.21	0.86	0.88	0.16	0.25	0.15	0.18	0.03	0.05	0.03	0.04	0.53	0.04	0.53	0.53
	**5 LAYER NN**	0.87	0.21	0.86	0.89	0.16	0.25	0.14	0.18	0.03	0.05	0.03	0.04	0.53	0.04	0.53	0.53
**90-D Readmission**	**AB**	0.87	0.001	0.86	0.87	0.58	0.001	0.57	0.58	0.08	0.000	0.081	0.083	0.73	0.14	0.001	0.14
	**Xg Boost**	0.77	0.001	0.77	0.78	0.69	0.001	0.68	0.69	0.003	0.000	0.003	0.003	0.73	0.01	0.000	0.01
	**LR**	0.78	0.01	0.75	0.81	0.41	0.02	0.38	0.44	0.06	0.001	0.05	0.06	0.60	0.10	0.001	0.10
	**RF**	0.75	0.01	0.72	0.77	0.46	0.02	0.43	0.50	0.10	0.001	0.09	0.10	0.61	0.16	0.001	0.16
	**SVM**	0.62	0.03	0.61	0.63	0.62	0.03	0.60	0.63	0.08	0.01	0.08	0.08	0.61	0.02	0.60	0.62
	**1 LAYER NN**	0.87	0.20	0.86	0.88	0.16	0.24	0.15	0.18	0.05	0.06	0.05	0.06	0.53	0.04	0.53	0.53
	**5 LAYER NN**	0.86	0.20	0.85	0.87	0.16	0.25	0.14	0.17	0.05	0.07	0.05	0.06	0.53	0.04	0.53	0.53

*LR* Logistic regression, *AB* Adaptive boosting, *Xg Boost* gradient boosting, *RF* Random forest, *SVM* Support vector machine, *NN* a 1-layer neural network and a 5 layered NN

**Table 5:** Testing results from 15% of the untrained dataset

		**Accuracy**				**Recall**				**Precision**				**AUROC**			
**Outcome**	**Model**	**M**	**SD**	**LB**	**UB**	**M**	**SD**	**LB**	**UB**	**M**	**SD**	**LB**	**UB**	**M**	**SD**	**LB**	**UB**
**90-D Composite Complications**	**AB**	0.87	.006	0.87	0.87	0.58	0.001	0.58	0.58	0.08	0.001	0.08	0.08	0.72	0.001	0.72	0.73
	**Xg Boost**	0.76	0.003	0.75	0.76	0.67	0.002	0.67	0.67	0.05	0.001	0.05	0.05	0.72	0.001	0.71	0.72
	**LR**	0.88	0.001	0.88	0.88	0.57	0.001	0.57	0.57	0.09	0.001	0.09	0.09	0.73	0.000	0.72	0.73
	**RF**	0.87	0.001	0.87	0.88	0.59	0.001	0.59	0.59	0.09	0.001	0.08	0.09	0.73	0.001	0.73	0.74
	**SVM**	0.63	0.01	0.63	0.63	0.61	0.01	0.61	0.61	0.03	0.001	0.03	0.03	0.62	0.01	0.62	0.62
	**1 Layer NN**	0.88	0.21	0.87	0.89	0.23	0.30	0.21	0.25	0.04	0.07	0.03	0.04	0.56	0.08	0.56	0.57
	**5 Layer NN**	0.88	0.21	0.87	0.90	0.23	0.30	0.21	0.25	0.04	0.07	0.03	0.04	0.56	0.08	0.56	0.57
**30-D Mortality**	**AB**	0.74	0.001	0.74	0.75	0.76	0.002	0.75	0.76	0.00	0.001	0.00	0.00	0.75	0.001	0.75	0.75
	**Xg Boost**	0.95	0.000	0.95	0.95	0.40	0.001	0.40	0.41	0.01	0.001	0.01	0.01	0.68	0.001	0.68	0.68
	**LR**	0.78	0.001	0.77	0.78	0.78	0.001	0.78	0.79	0.00	0.001	0.00	0.00	0.59	0.001	0.59	0.59
	**RF**	0.82	0.01	0.80	0.83	0.35	0.01	0.34	0.37	0.06	0.001	0.06	0.07	0.78	0.001	0.77	0.78
	**SVM**	0.66	0.09	0.66	0.67	0.74	0.05	0.74	0.74	0.00	0.001	0.00	0.00	0.70	0.04	0.70	0.70
	**1 Layer NN**	0.89	0.22	0.88	0.90	0.35	0.38	0.32	0.37	0.00	0.01	0.00	0.00	0.62	0.14	0.61	0.63
	**5 Layer NN**	0.89	0.22	0.88	0.91	0.34	0.38	0.32	0.36	0.00	0.01	0.00	0.00	0.62	0.15	0.61	0.63
**30-D Readmission**	**AB**	0.85	0.001	0.85	0.85	0.32	0.001	0.32	0.33	0.07	0.00	0.07	0.07	0.60	0.001	0.59	0.60
	**Xg Boost**	0.78	0.01	0.76	0.80	0.41	0.02	0.37	0.44	0.06	0.001	0.06	0.06	0.60	0.002	0.60	0.60
	**LR**	0.80	0.001	0.80	0.81	0.40	0.001	0.39	0.40	0.11	0.001	0.11	0.11	0.61	0.001	0.61	0.61
	**RF**	0.61	0.002	0.61	0.62	0.62	0.002	0.61	0.62	0.08	0.001	0.08	0.08	0.62	0.001	0.61	0.62
	**SVM**	0.62	0.03	0.61	0.63	0.60	0.01	0.60	0.61	0.05	0.003	0.05	0.05	0.61	0.02	0.60	0.62
	**1 Layer NN**	0.87	0.21	0.86	0.88	0.16	0.24	0.14	0.17	0.03	0.03	0.03	0.03	0.53	0.03	0.52	0.53
	**5 Layer NN**	0.87	0.21	0.86	0.89	0.15	0.24	0.14	0.17	0.03	0.05	0.03	0.03	0.53	0.03	0.52	0.53
**90-D Readmission**	**AB**	0.75	0.01	0.72	0.77	0.47	0.02	0.43	0.50	0.10	0.001	0.09	0.10	0.61	0.002	0.61	0.62
	**Xg Boost**	0.87	0.001	0.87	0.87	0.58	0.001	0.58	0.58	0.08	0.00	0.08	0.08	0.70	0.001	0.70	0.71
	**LR**	0.76	0.003	0.75	0.76	0.67	0.002	0.67	0.67	0.05	0.00	0.05	0.05	0.71	0.002	0.71	0.71
	**RF**	0.88	0.001	0.88	0.88	0.57	0.001	0.57	0.57	0.09	0.00	0.09	0.09	0.72	0.001	0.71	0.72
	**SVM**	0.60	0.03	0.59	0.62	0.61	0.03	0.60	0.62	0.08	0.01	0.08	0.08	0.61	0.02	0.60	0.62
	**1 Layer NN**	0.86	0.20	0.84	0.87	0.16	0.24	0.14	0.17	0.05	0.06	0.05	0.05	0.53	0.03	0.52	0.53
	**5 Layer NN**	0.86	0.20	0.85	0.87	0.15	0.24	0.14	0.17	0.05	0.06	0.05	0.05	0.53	0.03	0.52	0.53

*LR* Logistic regression, *AB* Adaptive boosting, *Xg Boost* gradient boosting, *RF* Random forest, *SVM* Support vector machine, *NN* a 1-layer neural network and a 5-layered NN

### External validation

External validation of model performance at our primary arthroplasty center showed the Adaptive Boost had the greatest performance for 90-day composite complications (AUROC 0.69; 95CI 0.68–0.69) (Table 6). Random Forest classifier was best at predicting 30-day mortality (AUROC 0.72; 95CI 0.72–0.73) and 30-day readmission (AUROC 0.68; 95 CI 0.67–0.68). Additionally, the Adaptive Boost classifier was the strongest model for the prediction of 90-day readmission (AUROC 0.72; 95 CI 0.72–0.73).

**Table 6:** External validation of 2% of untrained data

		**Accuracy**				**Recall**				**Precision**				**AUROC**			
**Outcome**	**Model**	**M**	**SD**	**LB**	**UB**	**M**	**SD**	**LB**	**UB**	**M**	**SD**	**LB**	**UB**	**M**	**SD**	**LB**	**UB**
**90-D Composite Complications**	**AB**	0.90	0.003	0.89	0.91	0.47	0.004	0.46	0.48	0.07	0.002	0.06	0.07	0.69	0.002	0.68	0.69
	**Xg Boost**	0.79	0.003	0.78	0.79	0.56	0.005	0.55	0.57	0.00	0.000	0.00	0.00	0.68	0.002	0.67	0.68
	**LR**	0.85	0.01	0.83	0.87	0.34	0.006	0.32	0.35	0.08	0.003	0.07	0.08	0.60	0.003	0.60	0.61
	**RF**	0.85	0.003	0.84	0.86	0.36	0.006	0.35	0.37	0.12	0.002	0.11	0.12	0.62	0.002	0.61	0.62
	**SVM**	0.74	0.01	0.74	0.75	0.59	0.02	0.59	0.60	0.03	0.001	0.03	0.03	0.67	0.01	0.67	0.67
	**1 Layer NN**	0.90	0.21	0.88	0.91	0.21	0.27	0.19	0.22	0.04	0.08	0.03	0.04	0.56	0.07	0.55	0.56
	**5 Layer NN**	0.90	0.21	0.88	0.91	0.20	0.27	0.19	0.22	0.04	0.11	0.03	0.04	0.55	0.07	0.55	0.56
**30-D Mortality**	**AB**	0.81	0.01	0.80	0.82	0.56	0.006	0.55	0.57	0.04	0.001	0.04	0.04	0.70	0.002	0.69	0.70
	**Xg Boost**	0.80	0.02	0.80	0.80	0.60	0.005	0.59	0.61	0.00	0.000	0.00	0.00	0.62	0.002	0.62	0.63
	**LR**	0.65	0.01	0.63	0.66	0.60	0.008	0.58	0.62	0.05	0.001	0.05	0.05	0.63	0.002	0.63	0.63
	**RF**	0.67	0.01	0.66	0.68	0.59	0.01	0.58	0.60	0.08	0.001	0.08	0.08	0.72	0.002	0.71	0.72
	**SVM**	0.77	0.11	0.76	0.77	0.59	0.12	0.58	0.60	0.00	0.001	0.00	0.00	0.68	0.05	0.68	0.68
	**1 Layer NN**	0.90	0.21	0.89	0.92	0.27	0.34	0.25	0.29	0.01	0.02	0.00	0.01	0.59	0.12	0.58	0.60
	**5 Layer NN**	0.90	0.22	0.89	0.92	0.27	0.34	0.25	0.29	0.01	0.04	0.00	0.01	0.59	0.12	0.58	0.59
**30-D Readmission**	**AB**	0.90	0.004	0.90	0.91	0.48	0.01	0.47	0.49	0.07	0.002	0.07	0.08	0.48	0.0002	0.48	0.48
	**Xg Boost**	0.96	0.000	0.96	0.96	0.00	0.000	0.000	0.001	0.00	0.000	0.00	0.00	0.62	0.002	0.62	0.63
	**LR**	0.87	0.003	0.87	0.88	0.36	0.01	0.35	0.37	0.09	0.002	0.09	0.09	0.61	0.002	0.61	0.62
	**RF**	0.86	0.004	0.85	0.87	0.34	0.01	0.33	0.35	0.12	0.003	0.12	0.13	0.68	0.002	0.67	0.68
	**SVM**	0.67	0.04	0.65	0.68	0.59	0.02	0.59	0.60	0.05	0.004	0.05	0.05	0.63	0.02	0.63	0.64
	**1 Layer NN**	0.88	0.87	0.90	0.88	0.16	0.24	0.15	0.18	0.04	0.06	0.04	0.05	0.53	0.04	0.53	0.54
	**5 Layer NN**	0.88	0.21	0.87	0.90	0.16	0.25	0.15	0.18	0.04	0.06	0.04	0.04	0.53	0.04	0.53	0.54
**90-D Readmission**	**AB**	0.93	0.001	0.92	0.93	0.42	0.004	0.41	0.43	0.08	0.001	0.08	0.08	0.72	0.002	0.72	0.73
	**Xg Boost**	0.83	0.001	0.82	0.83	0.62	0.003	0.62	0.63	0.00	0.000	0.00	0.01	0.61	0.003	0.60	0.62
	**LR**	0.82	0.01	0.80	0.84	0.38	0.02	0.35	0.42	0.07	0.001	0.07	0.07	0.62	0.003	0.62	0.63
	**RF**	0.80	0.01	0.78	0.82	0.43	0.01	0.40	0.46	0.10	0.002	0.09	0.10	0.69	0.002	0.68	0.69
	**SVM**	0.65	0.04	0.63	0.67	0.63	0.04	0.61	0.64	0.08	0.01	0.07	0.08	0.64	0.02	0.63	0.65
	**1 Layer NN**	0.87	0.20	0.86	0.89	0.16	0.24	0.14	0.17	0.06	0.07	0.05	0.06	0.53	0.04	0.53	0.54
	**5 Layer NN**	0.87	0.20	0.86	0.89	0.16	0.25	0.14	0.17	0.06	0.07	0.05	0.06	0.53	0.04	0.53	0.54

*LR* Logistic regression, *AB* Adaptive boosting, *Xg Boost* gradient boosting, *RF* Random forest, *SVM* Support vector machine, *NN* a 1-layer neural network and a 5 layered NN

### Explanatory variables

Feature importance was assessed for the top 3 variables that contributed the strongest weight to the top-performing model. Model predictors for 30-day mortality were found to be consistent between the test and external validation set demonstrating patient age, diseases of the circulatory system, and length of hospitalization to be the most important attributes for the RF classifier. Additionally, the prediction of 30-day readmission found that age, length of hospital stay, and the Elixhauser Comorbidity Index were the strongest contributors for the random forest classifier for models in both subsets.

As there was a discrepancy between classifier performance for 90-day composite complications and 90-day readmissions between the test and external validation, both model aspects were described. For composite complications and 90-day readmissions, our test set demonstrated that RF classifier performed best, with hospital stay, patient age, and Elixhauser Comorbidity Index being the top features for both outcomes. However, upon external validation, the Adaptive Boost classifier had the strongest discriminative performance for 90-day-composite complications and readmission, with age, surgical blood loss, and hospital length of stay being the top predictors for model output.

## Discussion

The purpose of this study was to leverage the benefits of a relatively large and accurate SPARCS dataset to train ML models capable of achieving good discrimination (AUROC > 0.80) on an externally validated dataset representing our PAC. Specifically, our outcomes focused on 90-day complications, 30-day mortality, and 30- & 90-day readmissions. If successful, the study would demonstrate the ability to use “big” data to effectively predict single hospital system-level complications, mortality, and readmissions. This would not only benefit our hospital system but also other hospital systems in NYS. While the results showed that no ML model achieved an AUROC > 0.80, overall model performance was on par with similar studies and model performance doesn’t discredit relevant findings. Our results showed that the RF classifier had the strongest discriminative performance for 30-day mortality (AUROC = 0.72) and readmissions (30-day AUROC = 0.68) on our external validation set. For 90-day composite complication and 90-day readmissions, the Adaptive Boost classifier was the best predictor in our external validation set (AUROC = 0.69 and 0.72, respectively). While no ML model in the testing dataset achieved an AUROC > 0.78, the drop in performance between the best-performing ML model in the testing dataset and best-performing model in the external validation dataset was no more than 0.06 points on an AUROC. This finding is important as it speaks to the potential generalizability of the SPARCS dataset to any arthroplasty center located within NYS. To the best of our knowledge, this study represents the most rigorous ML analysis of the SPARCS database for potential use in TJA care.

Mohammed et al. used the National Inpatient Service (NIS) administrative database to internally validate four ML algorithms (LR, Xg Boost, RF classifier, and NN) to perform predictive modeling for discharge disposition, composite post-surgical complications, and the need for blood transfusion after TJA [16]. The group found that the Xg Boost was capable of predicting outcomes, with an AUROC of 0.80–0.87. While an impressive proof of concept, the lack of external validation of their NIS model leaves questions about the potential clinical utility of the NIS dataset at any given arthroplasty center. The NIS, although advantageous due to its quantity of data, was created to assess national trends and correlation and not to be used to potentially direct care at a statewide level. The database randomly samples 20% of hospitals nationwide, thus potentially leading to significant unintended bias as to where information is collected [21, 23]. Furthermore, there are no weights or classifiers applied to each state to account for the difference in the number of metrics collected [22]. Devana et al. investigated the use of the California Office of Statewide Health Planning and Development state dataset (OSHPD) to train and test ML algorithms to predict complications following TKA [24]. The group trained ML models (LR, Xg Boost, Adaptive Boost, RF classifier, and ensemble) on 156,750 TKA patients and demonstrated that Adaptive Boost had the best discriminative performance with an AUROC of 0.68, being congruent to the findings of our external validation model. It is important to note that both studies, by Mohammed et al. and Devana et al., relied on internal validation of their respective datasets. Therefore, while the results of these studies provide valuable insights into the potential predictive capabilities of ML algorithms for TJA outcomes, external validation on diverse datasets is essential to the establishment of the reliability and applicability of these models in real-world clinical settings. External validation helps mitigate the concerns of dataset-specific biases and increases confidence in the generalizability of the findings, thus enhancing the overall clinical utility of the developed models.

Several studies have assessed differences in the model prediction of neural networks in comparison to more traditional ML algorithms [25, 26]. They tried to answer the question: “Would these more complex ML algorithms outperform their less advanced counterparts”. However, most of these studies failed to incorporate crucial detail as to the layer of models used in their neural network. In fact, few studies have assessed whether a neural network with an increased number of hidden layers would improve model performance [27, 28]. A neural network at its most basic form has just one layer of inputs, one layer of active units, and one layer of outputs. The outputs do not interact, so a network with “n” outputs can be treated as “n” separate single-output networks. Therefore, a single-layer neural network can only be used to represent linearly separable functions. However, multi-layer networks can learn to develop interconnections and unforeseen associations around examples in some high-dimensional space that can separate and classify them, thereby overcoming the limitation of linear separability. Our study found that the 1- and 5-layer neural networks were nearly identical in their performance regarding AUROC and were inferior to the other models when predicting the TJA outcomes of interest. The reasons for decreased performance of our NN are not entirely clear but it does provide evidence that highly complex ML algorithms do not necessarily confer improved performance in the SPARCS dataset. Total joint arthroplasty has witnessed a notable shift towards value-based care, which emphasizes the delivery of high-quality, cost-effective, and patient-centered healthcare [10, 28]. This shift has been driven by the need to improve patient outcomes, control healthcare costs, and enhance overall value in the TJA field. Risk calculators through ML algorithms may accurately predict factors that may pose a higher risk for poor outcomes following TJA. These calculators have the potential to be utilized through a spectrum of preoperative care to the point of discharge from the site of surgical service. However, it is important to validate these models externally to assess their generalizability to other healthcare centers. Therefore, future studies in machine learning should prioritize external validation of ML models to ensure their reliability and effectiveness. One of the primary goals of this study was to ensure external validation of the models which is frequently missing in orthopaedic literature assessing ML model performance. The failure of external validation of such models may lead to misleading conclusions. For example, our results showed a discrepancy in model performance between the test and external validation sets. Xg Boost was the best performing model for predicting 90-day readmission in our test set, while the AB model performed better for our PAC data. Given the implications of wrong predictions in patient care, there must be continued emphasis on external validation for future AI-based investigations.

This study is not without limitations and the limitations are primarily present in the dataset. SPARCS does have the limitation of being a payor-based dataset which, to some extent, limits the validity of the clinical markers (e.g., identification of acute and chronic conditions). However, one needs to trade that off with its many strengths as previously mentioned. Another possible limitation may be due to the possible unique separation in population demographics of New York City when compared to the rest of NYS. As such, risk calculators created by SPARCS-trained data may not execute consistently on a PAC center. Further research should be conducted to identify the predictive value that ML can have on assessing TJA in New York City patients when trained with an NYS statewide dataset. Finally, the SPARCS dataset included the years 2012 through 2016, which contained the evolution of practice pattern changes driven by the CJR model and other bundled payments. These bundle payments are known to be associated with decreased complications and readmissions. Future studies with more recent data may affect future model performance and conclusions.

## Conclusions

This study was the first to investigate the use of ML to create a predictive risk calculator from a highly validated statewide database and externally validate it to a single PAC within the same geographic region. All models showed low to moderate discrimination on an AUROC, which is consistent with recent model performance in the TJA literature. However, this study included external validation performance which is lacking in many prior studies. Furthermore, the external validation performance was of moderate discrimination on an AUROC. More advanced NN models did not perform better than less sophisticated ML models. The importance of detailing the dataset, model construction, and model validation cannot be overstated. The unique composition of New York City and its subsequent influence on future ML risk calculators created through SPARCS is a potential area of investigation.

## Data Availability

Our application to acquire NYS SPARCS data does not allow sharing of data to those outside of the IRB.
